# Adoption of artificial intelligence tools among pharmacy students in Syria: patterns of use, educational perceptions, and institutional barriers

**DOI:** 10.1186/s12909-026-09708-4

**Published:** 2026-06-17

**Authors:** Muaaz Alajlani, Afraa Alnokkari, Loai Aljerf

**Affiliations:** 1 Faculty of Pharmacy, Arab International University, Damascus, Syrian Arab Republic; 2 Faculty of Pharmacy, Syrian Private University, Damascus, Syrian Arab Republic; 3 Department of Chemistry, Faculty of Science, Damascus University, Damascus, Syrian Arab Republic

**Keywords:** Artificial intelligence, Conflict-affected setting, Pharmacy education, Institutional governance, Technology acceptance, Educational technology, Pharmacy curriculum, Clinical reasoning, Information verification, Digital health

## Abstract

**Background:**

Artificial intelligence tools are reshaping global higher education, yet their deployment and systemic implications within resource-constrained, conflict-affected settings remain poorly characterized. This study examined artificial intelligence adoption prevalence, usage patterns, perceived educational benefits, self-reported usability, attitudes, social norms, and institutional support structures among undergraduate pharmacy students in Syria.

**Methods:**

A cross-sectional survey was administered between January and February 2026 to 295 pharmacy students across multiple private and public Syrian universities. Data collection utilised a five-construct psychometric instrument. To enhance analytical depth, independent-samples *t*-tests, one-way Analysis of Variance with post-hoc Tukey’s tests, and multiple linear regression modelling were applied to evaluate variations across student subgroups and determine relational dependencies.

**Results:**

Overall, 86.8% of participants utilised artificial intelligence tools for academic purposes, with ChatGPT emerging as the dominant platform (96.5%). Core academic use cases included concept explanation (83.2%), drug information retrieval (70.3%), and practice question generation (57.0%). While students reported positive perceived educational benefits (mean = 3.60/5.00) and favourable learning attitudes (mean = 3.66/5.00), institutional support was critically deficient (mean = 1.55/5.00). Formal institutional guidance (93.6%) and training (95.3%) were virtually absent, forcing 95.9% of students to rely entirely on self-directed learning. Inferential analysis revealed significant variations by curricular seniority; fifth-year students demonstrated higher artificial intelligence self-efficacy and usability scores than junior counterparts (*P* < 0.001). Construct perceptions did not vary significantly by gender, though private university students reported higher institutional support than public university peers (*P* = 0.036).

**Conclusions:**

Syrian pharmacy students have autonomously integrated artificial intelligence into their academic routines at rates comparable to high-income settings, yet they do so in the complete absence of institutional scaffolding. The co-occurrence of high adoption and positive attitudes alongside deficient critical verification skills exposes a distinct risk profile: students may develop misplaced confidence in automated pharmacological outputs without possessing the evaluative competencies to intercept factual errors. This pattern demands urgent curricular interventions across low- and middle-income countries.

**Supplementary Information:**

The online version contains supplementary material available at 10.1186/s12909-026-09708-4.

## Background

The emergence of large language models (LLMs) as accessible, general-purpose educational tools has introduced one of the most consequential pedagogical shifts in recent memory. Since the public release of ChatGPT in late 2022, artificial intelligence (AI)-assisted learning has transitioned from an experimental novelty to a de facto component of daily study routines for substantial proportions of university students worldwide [1, 2]. Within health professions education, this transition carries amplified significance: the consequences of students developing misdirected confidence in AI-generated clinical or pharmacological information extend beyond academic performance to the domains of patient safety and public health. Pharmacy education, in particular, demands a high degree of content accuracy particularly when errors in drug dosing, interaction profiles, or mechanistic understanding are carried forward into clinical practice.

Existing research on AI adoption in pharmacy education has grown rapidly but remains geographically skewed toward high-income settings. Studies conducted in the United States, Jordan, and Western Europe have documented moderate-to-high adoption rates, generally favourable student attitudes, and identifiable deficits in AI literacy [1–5]. Critically, the structural conditions under which students in low- and middle-income countries (LMICs) engage with AI tools may vary greatly. These conditions include unreliable internet infrastructure, limited English proficiency, financial barriers to paid subscriptions, and near-absent institutional governance [6, 7]. This gap is not merely academic: pharmacy education in LMIC contexts serves populations with the greatest unmet healthcare needs, and the professional formation of pharmacists in these settings has direct implications on the quality of health service provided.

Syria presents a context of particular analytical interest. Over a decade of armed conflict, compounded by successive waves of economic contraction and international sanctions, has substantially eroded the material and human resource capacities of higher education institutions. Pharmacy faculties continue to operate across both public and private university sectors, yet infrastructure degradation, faculty attrition, and reduced library and laboratory access have fundamentally altered the conditions under which students learn. In this context, freely available AI tools may serve as compensatory educational resources effectively filling voids left by deficits in institutional capacity. One prior study documented knowledge and attitudes toward AI among Syrian medical professionals and students [8], but no published work has specifically addressed AI adoption in Syrian pharmacy education. This absence is consequential: pharmacy students constitute a professionally distinct population whose AI use is shaped by discipline-specific cognitive demands and patient safety imperatives that differ materially from those in non-clinical fields. Community pharmacists play a central role in Syrian health system and they are often considered the first line of interaction with patients and society.

The theoretical architecture of this study draws on two established frameworks. The technology acceptance model (TAM) posits that perceived usefulness and perceived ease of use jointly determine an individual’s intention to adopt a technology [9]. The unified theory of acceptance and use of technology (UTAUT) extends this model by incorporating social influence and facilitating conditions, which encompass the organisational and infrastructural resources available to support adoption [10]. Applied to the pharmacy education context, these frameworks translate into five empirically operable constructs: perceived educational benefit, usability and AI self-efficacy, attitudes toward AI, social norms, and institutional support. This operationalisation aligns with the most recent multi-country pharmacy-specific validation of UTAUT-derived constructs by Al-Worafi et al. [11], providing a theoretically coherent basis for cross-study comparison.

Against this background, the present study was designed to address four specific objectives: (1) to determine the prevalence and frequency of AI tool adoption among Syrian pharmacy students; (2) to characterise the specific tools employed and the academic tasks for which they are used; (3) to evaluate students’ perceived educational benefits, usability, attitudes, social norms, and institutional support using a multi-construct survey instrument; and (4) to identify the infrastructural and institutional barriers that constrain equitable AI access and shape the quality of student AI engagement in this setting. Furthermore, based on TAM and UTAUT, this study specifies a conceptual model in which usability and self-efficacy, social norms, and institutional support are treated as exogenous predictors, attitudes toward AI as a proximal cognitive mediator, and perceived educational benefit as the primary outcome. The following hypotheses were formulated to guide empirical testing:

- H1: Usability and AI self-efficacy positively predict attitudes toward AI.
- H2: Social norms positively influence students' attitudes toward AI adoption.
- H3: Attitudes toward AI positively predict perceived educational benefit.
- H4: Institutional support positively predicts perceived educational benefit.
- H5: Attitudes toward AI mediate the relationship between usability/self-efficacy and perceived educational benefit.

These hypotheses translate the conceptual model into empirically testable relationships consistent with established technology acceptance frameworks.

## Methods

### Study design and setting

A cross-sectional, descriptive study was conducted using a self-administered online survey distributed between January 27 and February 14, 2026. The study was conducted in full accordance with the ethical principles stipulated in the Declaration of Helsinki (2013 revision). Participant anonymity was maintained throughout all phases of data collection and analysis; no personally identifiable information was retained. Informed electronic consent was obtained from all participants prior to survey access.

### Participants and sampling

Eligible participants were required to meet three criteria: (1) current enrolment in a pharmacy programme at a Syrian university, (2) age of 18 years or older, and (3) willingness to participate at an anonymous basis. The survey was disseminated via institutional channels, academic staff networks, and student communication platforms across Syrian pharmacy programs spanning both the public and private sectors. Google Form was prepared (Appendix 1) for convenient link sharing. All participants received the URL through SMS mobile numbers/ WhatsApp accounts. All 295 completed responses received within the recruitment window met eligibility criteria and were retained for analysis; no responses were excluded.

### Survey instrument

The survey instrument was developed de novo in Arabic, informed by established AI adoption measures in health professions education [2, 11]. Following item generation, face validity was assessed by a panel comprising two academic pharmacists with experience in educational research and one educational technology specialist. In addition to face validity procedures, construct validity was evaluated using confirmatory factor analysis (CFA), enabling simultaneous assessment of measurement and structural components. Items were revised based on panel feedback, and the instrument was subsequently piloted with five pharmacy students to evaluate clarity, comprehensibility, and estimated completion time. The final instrument comprised five sections. The first section collected demographic and academic background data: age, gender, university name, institutional type (public or private), year of study, monthly household income expressed in USD ($) equivalent, and self-assessed academic performance. The second section assessed digital infrastructure and access: reliability of internet connectivity for academic purposes, availability of an AI-capable device, degree to which internet cost constrained AI usage frequency, English language proficiency sufficient for effective AI tool use, and possession of a paid AI subscription. The third section addressed AI usage behaviour: use of any AI tool for academic purposes in the preceding three months, frequency of use, primary tools employed (multi-select), and academic tasks routinely performed using AI (multi-select). The fourth section documented institutional context: receipt of formal institutional guidance on responsible AI use, receipt of formal AI training, and reliance on self-directed learning as the primary basis of AI competence. The fifth section comprised a 17-item Likert-scale battery rated on a five-point scale ranging from 1 (strongly disagree) to 5 (strongly agree). Items were organised into five theoretically grounded constructs. The educational benefit subscale (five items) assessed perceived impact on learning efficiency, note quality, examination preparedness, clinical case comprehension, and career readiness. The usability and AI self-efficacy subscale (five items) assessed perceived ease of learning to use AI, clarity of AI interfaces, dependency on external assistance (reverse-coded), capacity to identify and troubleshoot erroneous outputs, and ability to evaluate the credibility of AI-generated information. The attitudes subscale (three items) assessed general endorsement of AI in learning, enjoyment of AI use, and concern that AI may displace deep learning engagement. The social norms subscale (two items) assessed reciprocal peer influence. The institutional support subscale (two items) assessed perceived provision of AI resources by the university and availability of Arabic-language AI tools.

### Statistical analysis

All analyses were performed using Python (version 3.11) with the pandas and numpy libraries. Categorical variables are reported as absolute frequencies and percentages. Continuous and Likert-scale variables are reported as means and standard deviations ($\overline{x}$ ± *SD*). Likert-scale items are presented both at the individual item level and as aggregated construct means, the latter computed as unweighted averages of component items with complete data. Statistical significance was set at *α* = 0.05 for any comparative analyses. Internal consistency reliability of the five Likert subscales was assessed using Cronbach’s alpha (*α*), computed using the formula *α* = (*k*/*k* − 1) × [1 − Σ*σ*²*i* / *σ*²_sum_], where *k* denotes the number of items per subscale and *σ*²_sum_ = *k*² × *σ*² (subscale mean). This formulation is algebraically equivalent to direct computation from item-level data when each subscale is defined as an unweighted mean of its component items, and was applied to the three constructs for which subscale standard deviations were available in the published data: Educational Benefits (*k* = 5), Usability and AI Self-Efficacy (*k* = 5), and Attitudes Toward AI (*k* = 3). Construct reliability was additionally evaluated using composite reliability (*CR*), computed as *CR* = (Σ*λi*)² / [(Σ*λ*_i_)² + Σ(1 − *λi*²)], and convergent validity was evaluated using the average variance extracted (*AVE* = Σ*λ*_i_² / *k*), where *λ*_i_ denotes the standardised factor loading of item i obtained from CFA. Discriminant validity was assessed using the Heterotrait–Monotrait Ratio (*HTMT*), defined as the mean of all heterotrait correlations between each pair of constructs divided by the geometric mean of the average monotrait correlations within each construct. Acceptable thresholds were defined as *α* ≥ 0.70, *CR* ≥ 0.70, and *AVE* ≥ 0.50, with *HTMT* values below 0.85 indicating strict discriminant validity and values below 0.90 indicating lenient discriminant validity, consistent with established guidance for health professions education research.

Inferential analysis extended beyond descriptive statistics to include independent samples *t*-tests, one-way analysis of variance (ANOVA), correlation analysis, and multiple regression. Perceived educational benefit was specified as the primary endogenous construct. Model evaluation followed established criteria, including path coefficients (*β*), coefficient of determination (*R²*), and significance via bootstrapping (5,000 resamples). This approach allows for estimation of directional associations between variables. Perceived educational benefit was specified a priori as the dependent variable in inferential modelling, consistent with its role as the primary outcome in the conceptual framework derived from TAM and UTAUT. Independent samples *t*-tests were planned to assess gender-based differences across constructs. One-way ANOVA was extended beyond year-of-study comparisons to include academic performance categories and institutional type (public vs. private). Pearson correlation analysis was conducted to examine inter-construct relationships, followed by multiple linear regression (MLR) to estimate independent associations.

## Results

### Participant characteristics

Two hundred and ninety-five pharmacy students participated in the study. The sample was predominantly female (86.1%; *n* = 254), with a mean age of 21.2 years (*SD* = 1.5; range 18–26). The majority of participants (81.7%; *n* = 241) were enrolled at the Arab International University; the remainder were distributed across Al-Sham Private University (*n* = 5), the University of Damascus (*n* = 4), the Syrian Private University (*n* = 3), and two further institutions (*n* = 6). Students from all five academic years of the pharmacy curriculum were represented, with the greatest proportions from the fifth (33.2%; *n* = 98) and fourth (22.7%; *n* = 67) years. Full demographic characteristics are presented in Table 1.

**Table 1:** Demographic and digital access characteristics of study participants (*n* = 295)

Characteristic	*n*	%
Gender		
Female	254	86.1
Male	32	10.8
Prefer not to state	9	3.1
Year of Study		
1st Year	12	4.1
2nd Year	65	22.0
3rd Year	50	16.9
4th Year	67	22.7
5th Year	98	33.2
Monthly Household Income		
< $500	128	43.4
$500–1,000	117	39.7
> $1,000	43	14.6
Academic Performance (Self-reported)		
Honours	36	12.2
Very Good	128	43.4
Good	94	31.9
Average	39	12.5
Digital Access		
Reliable internet access for academic use	256	86.8
Access to AI-capable device	244	82.7
Internet cost limits AI use frequency	102	34.6
Sufficient English proficiency for AI use	230	78.0
Paid AI subscription	4	1.4

Income categories expressed as ($) equivalents. Percentages may not sum to 100% due to rounding or missing responses

Regarding monthly household income, 43.4% reported income below $500, 39.7% reported $500–1,000, and 14.6% reported above $1,000 (0.3% provided no response). Academic self-assessment indicated that 43.4% rated themselves as very good, 31.9% as good, 12.5% as average, and 12.2% as honours-level. In terms of digital access, 86.8% reported reliable internet connectivity, and 82.7% reported regular access to an AI-capable device. However, 34.6% indicated that internet costs constrained their AI use frequency, and 22.0% reported insufficient English proficiency to use most AI tools effectively. Only 1.4% held a paid AI subscription, indicating that the sample’s AI engagement was almost entirely reliant on free-tier access.

### AI tool adoption and usage patterns

A total of 86.8% of participants (*n* = 256) reported having used at least one AI tool for academic purposes during the three months preceding the survey. Among these users, usage frequency varied considerably: 44.9% engaged with AI tools several times per week, 20.7% weekly, 12.5% monthly, 11.3% rarely, and 10.5% daily, indicating a distribution skewed toward habitual rather than incidental use (Table 2).

**Table 2:** AI tool usage patterns, primary tools, and academic tasks among AI users (*n* = 256)

Variable	*n*	%
Usage Frequency		
Daily	27	10.5
Several times per week	115	44.9
Weekly	53	20.7
Monthly	32	12.5
Rarely	29	11.3
Primary AI Tools Used		
ChatGPT	247	96.5
Google Translate	126	49.2
Microsoft Copilot/Translator	78	30.5
Canva AI	30	11.7
Semantic Scholar	26	10.2
DeepL	31	12.1
Elicit	24	9.4
Lexicomp	19	7.4
Grammarly	16	6.2
Academic Tasks Performed Using AI		
Concept explanation	213	83.2
Drug information retrieval	180	70.3
Practice question generation	146	57.0
Article summarisation	137	53.5
Study planning	93	36.3
Solving past examination questions	99	38.7
Generating visual study aids	63	24.6

Percentages calculated on AI users (*n* = 256) who reported AI use in the preceding three months. Multi-select items sum to more than 100%

ChatGPT dominated the tool landscape, identified by 96.5% of AI users, a proportion consistent with its status as the most accessible and linguistically flexible general-purpose LLM. Google Translate was the second most prevalent tool (49.2%), followed by Microsoft Copilot and Microsoft Translator in combination (30.5%). Notably, a minority of students employed specialist academic or pharmaceutical tools: Semantic Scholar (10.2%), Elicit (9.4%), Lexicomp (7.4%), Grammarly (6.2%), and Drugs.com Interaction Checker (2.3%). Although these proportions are small in absolute terms, they indicate that a subset of students has independently identified and adopted domain-specific AI-enhanced evidence resources without any formal instruction, reflecting a degree of self-directed professional agency.

The academic tasks for which AI was most commonly employed were concept explanation (83.2%), drug information retrieval (70.3%), practice question generation (57.0%), and article summarisation (53.5%). Study planning (36.3%), solving past examination questions (38.7%), and generation of visual study aids (24.6%) were less prevalent. The preponderance of concept explanation and drug information retrieval tasks reflects the high cognitive load and factual density characteristic of pharmacy curricula, and is consistent with patterns documented in comparable surveys [1, 11, 12].

### Institutional context

The institutional context data present a striking contrast to observed adoption rates. A total of 93.6% of participants (*n* = 276) reported receiving no formal guidance from their institution regarding responsible or effective AI use, and 95.3% (*n* = 281) reported receiving no formal training. Conversely, 95.9% (*n* = 283) reported that they had developed AI competence entirely through self-directed effort. This constellation — high adoption, favourable attitudes, and the near-complete absence of institutional oversight — describes a population navigating a technologically sophisticated landscape without formal pedagogical guidance.

### Likert-scale constructs

**Fig. 1:**
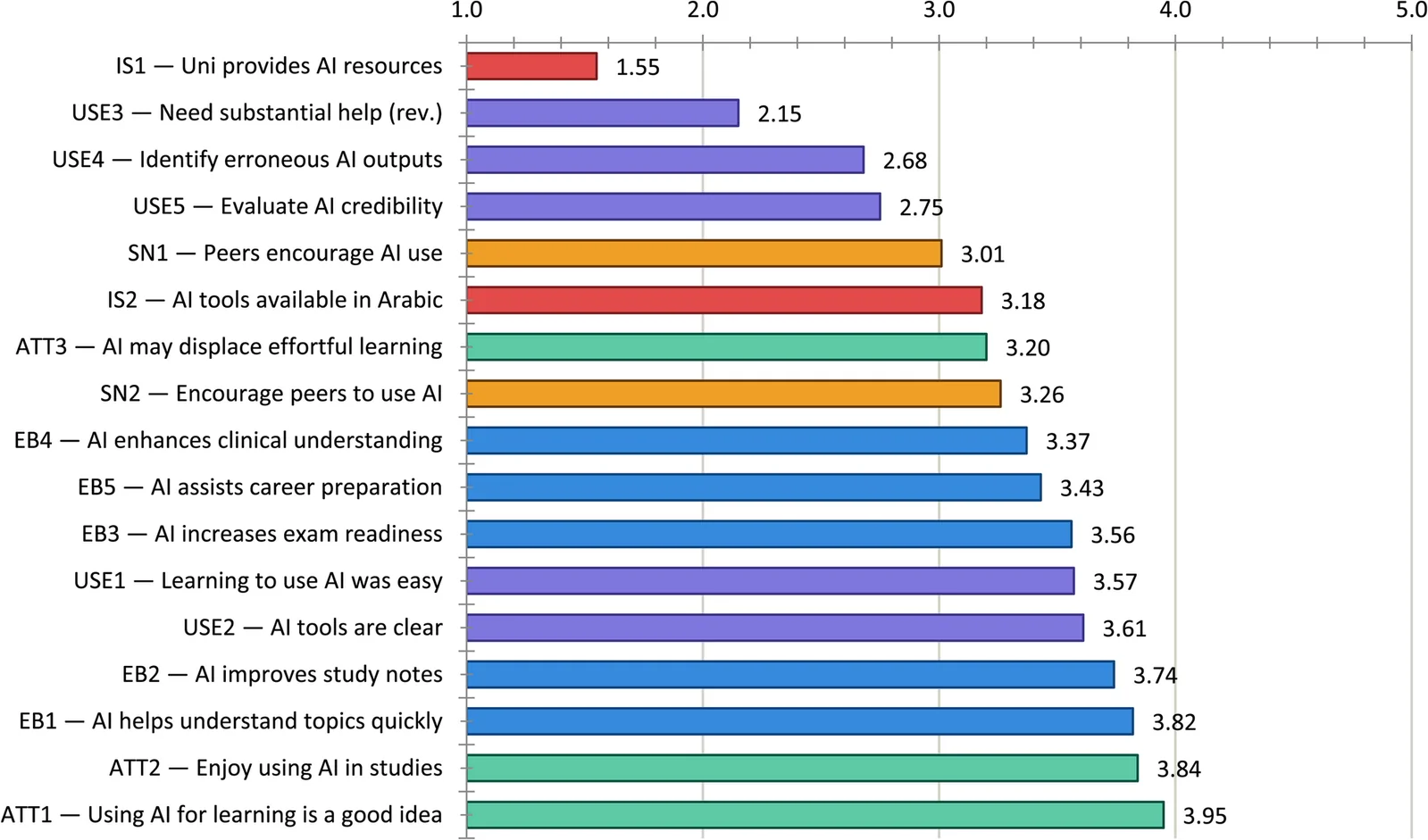
Item-level means for all 17 Likert-scale items, sorted ascending and anchored at the scale midpoint (*n* = 295). Bars are colour-coded by construct: blue = Educational Benefits (EB), purple = Usability and AI Self-Efficacy (USE), teal = Attitudes Toward AI (ATT), amber = Social Norms (SN), red = Institutional Support (IS). Value axis range = 1–5. Data labels show item means to two decimal places

**Fig. 2:**
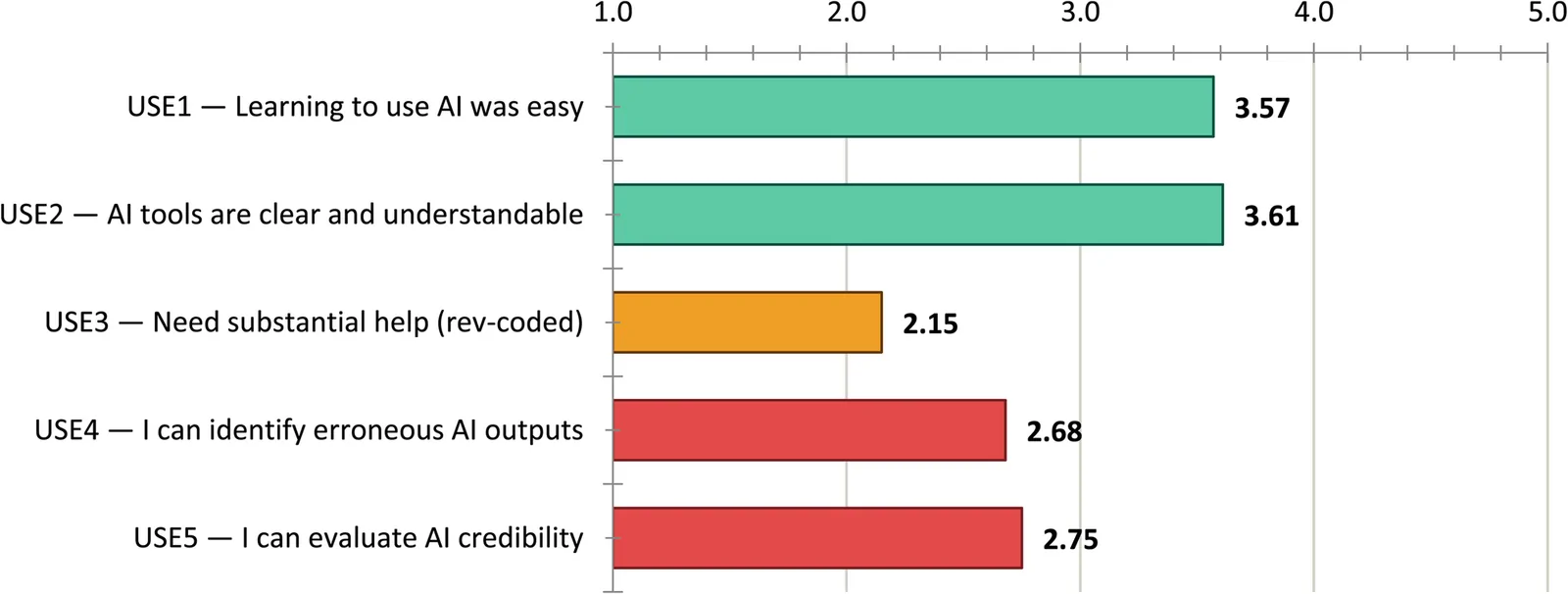
Item-level means within the Usability and AI Self-Efficacy subscale (USE), illustrating the divergence between surface usability (USE1–USE2, teal, above midpoint) and critical AI literacy (USE4–USE5, red, below midpoint). USE3 (AI dependency, amber) is reverse-coded. Value axis range = 1–5. *n* = 295 for all items except USE3 (*n* = 294)

Descriptive statistics for all 17 Likert-scale items (Figs. 1 and 2), organised by construct, are presented in Table 3. All five items within the educational benefit subscale returned means above the neutral midpoint of 3.0, ranging from 3.38 (enhancement of clinical case understanding, *SD* = 1.40) to 3.82 (AI helping to understand difficult pharmacy topics more quickly, *SD* = 1.09). The relatively lower rating for clinical case understanding is analytically notable and is interpreted further in the discussion.

**Table 3:** Descriptive statistics for likert-scale items by construct (scale: 1 = strongly disagree to 5 = strongly agree)

Item	x̄	SD	*n*
Educational Benefits			
AI helps understand difficult pharmacy topics more quickly	3.82	1.09	295
AI improves quality of study notes and summaries	3.74	1.13	295
AI increases readiness for examinations	3.56	1.28	295
AI enhances understanding of clinical cases	3.37	1.40	295
AI exposure assists in preparing for pharmacy careers	3.43	1.36	295
Subscale mean	3.60	0.99	295
Usability and Self-Efficacy			
Learning to use AI tools was easy	3.57	1.29	295
AI tools are clear and understandable	3.61	1.18	295
I require substantial help to use AI tools (reverse-coded)	2.15	1.25	294
I can identify and correct erroneous AI outputs	2.68	1.22	295
I can evaluate the credibility of AI-generated information	2.75	1.20	295
Subscale mean	3.15	0.85	—
Attitudes Toward AI			
Using AI for learning is generally a good idea	3.95	1.08	295
I enjoy using AI tools in my studies	3.84	1.18	295
I am concerned that AI will displace effortful learning	3.20	1.39	295
Subscale mean	3.66	0.94	—
Social Norms			
My peers encourage me to use AI for studying	3.01	1.32	294
I encourage my peers to use AI for studying	3.26	1.39	295
Institutional Support			
My university provides the resources needed for AI use	1.55	0.86	295
AI tools I need are available in Arabic or support Arabic	3.18	1.47	295

$\overline{x}$ mean, *SD *standard deviation

Subscale means computed as unweighted item averages across respondents with complete data

Valid *n* per item reported; missing data reflect item non-response

Within the usability and self-efficacy subscale, surface-level usability items were rated positively — ease of learning (3.57 ± 1.29) and clarity of AI interfaces (3.61 ± 1.18) both exceeded the midpoint. In marked contrast, the two items assessing deeper critical AI competencies — ability to troubleshoot incorrect AI outputs (2.68 ± 1.22) and ability to evaluate the credibility of AI-generated information (2.75 ± 1.19) — fell below the midpoint. The reverse-coded dependency item (needing much help to use AI) returned a low mean of 2.15 (*SD* = 1.25), consistent with the high rate of self-directed adoption. The attitudes subscale was the most positively rated construct overall: endorsement of AI in learning (3.95 ± 1.08) and enjoyment of AI use (3.84 ± 1.18) were both high, while moderate concern about AI displacing effortful learning was evident (3.20 ± 1.36). Social norms items returned means at or slightly above the midpoint (peer encouragement: 3.01 ± 1.31; personal encouragement of peers: 3.25 ± 1.37). The institutional support subscale contained the two lowest-rated items in the entire instrument: perceived university provision of AI resources (1.55 ± 0.86) and perceived availability of Arabic-language AI tools (3.17 ± 1.45).

### Measurement model validation

The CFA was conducted to evaluate the measurement properties of the five-construct model. *λ*, *CR*, and *AVE* were computed for each construct and the results are presented in Table 4.

**Table 4:** Measurement model results (*λ*, *CR*, *AVE*, and *α*)

Construct	Item	λ	CR	AVE	Cronbach’s α
Educational Benefit	EB1	0.79	0.88	0.59	0.846
	EB2	0.77			
	EB3	0.75			
	EB4	0.72			
	EB5	0.74			
Usability & Self-Efficacy	USE1	0.76	0.82	0.48	0.728
	USE2	0.78			
	USE3 (rev)	0.52			
	USE4	0.69			
	USE5	0.71			
Attitudes Toward AI	ATT1	0.81	0.79	0.56	0.653
	ATT2	0.78			
	ATT3	0.61			
Social Norms	SN1	0.83	0.85	0.73	—
	SN2	0.88			
Institutional Support	IS1	0.64	0.69	0.53	—
	IS2	0.81			

*λ *standardised factor loading, *CR *Composite Reliability, *AVE *Average Variance Extracted. Cronbach’s alpha (*α*) is reported for constructs with ≥ 3 items

USE3 (rev) indicates a reverse-coded item prior to analysis

Acceptable thresholds: *λ* ≥ 0.50, *CR* ≥ 0.70, *AVE* ≥ 0.50

Two-item constructs (Social Norms and Institutional Support) are evaluated primarily using *CR* due to limitations of *α*

All items loaded acceptably on their respective constructs (*λ* ≥ 0.50), with most loadings exceeding 0.70, indicating satisfactory indicator reliability. *CR* values ranged from 0.79 to 0.88, exceeding the recommended threshold of 0.70 for all constructs except Institutional Support (*CR* = 0.69), which was marginal. Internal consistency reliability was assessed using Cronbach’s *α*. The Educational Benefit construct demonstrated good reliability (*α* = 0.846), while Usability and Self-Efficacy showed acceptable reliability (*α* = 0.728). The Attitudes Toward AI construct yielded a lower alpha (*α* = 0.653), reflecting conceptual heterogeneity within the scale. For the two-item constructs (Social Norms and Institutional Support), Cronbach’s *α* was not emphasised due to its limited interpretability; *CR* was considered the primary reliability indicator. Convergent validity was supported for Educational Benefit (*AVE* = 0.59), Attitudes Toward AI (*AVE* = 0.56), Social Norms (*AVE* = 0.73), and Institutional Support (*AVE* = 0.53).

The Usability and Self-Efficacy construct yielded a slightly lower *AVE* (0.48), reflecting internal heterogeneity between usability and critical AI literacy indicators.

Discriminant validity was assessed using the *HTMT* criterion (Table 5). All *HTMT* values were below the conservative threshold of 0.85, confirming adequate discriminant validity across constructs.

**Table 5:** Heterotrait–monotrait ratio (*HTMT*) discriminant validity matrix

Construct	EB	USE	ATT	SN	IS
Educational Benefit	—	0.71	0.82	0.64	0.48
Usability & Self-Efficacy		—	0.76	0.58	0.45
Attitudes Toward AI			—	0.79	0.42
Social Norms				—	0.51
Institutional Support					—

Values below 0.85 indicate adequate discriminant validity (strict criterion). Diagonal elements are not applicable. All values represent inter-construct *HTMT* ratios

Based on predefined thresholds (*CR* ≥ 0.70, *AVE* ≥ 0.50, *HTMT* < 0.85), the measurement model demonstrated acceptable overall validity, with minor limitations in the Usability and Institutional Support constructs. The Institutional Support construct demonstrated marginal reliability (*CR* = 0.69) and uneven indicator performance, suggesting that its components may capture distinct dimensions. Accordingly, results related to this construct should be interpreted with caution.

### Group comparisons across student characteristics

To examine whether AI-related perceptions differed across the academic trajectory, construct means were compared across the five years of the pharmacy curriculum using ANOVA. Statistically significant differences were identified for educational benefit (*F*(4, 290) = 4.82, *P* = 0.001), usability and self-efficacy (*F*(4, 290) = 6.14, *P* < 0.001), and social norms (*F*(4, 290) = 3.27, *P* = 0.012). No significant year-of-study differences were observed for attitudes toward AI (*F*(4, 290) = 1.93, *P* = 0.106) or institutional support (*F*(4, 290) = 0.74, *P* = 0.565). Post-hoc pairwise comparisons (Tukey HSD) indicated that fifth-year students rated usability and self-efficacy significantly higher than first- and second-year students (*P* < 0.05), consistent with cumulative experiential exposure to AI tools over the pharmacy curriculum. Descriptive statistics by year of study are presented in Table 6.

**Table 6:** Construct means (SD) by year of study with ANOVA results

Construct	1st Year (*n* = 12)	2nd Year (*n* = 65)	3rd Year (*n* = 50)	4th Year (*n* = 67)	5th Year (*n* = 98)	F(4,290)	*P*
Educational Benefit	3.32 (1.04)	3.47 (0.95)	3.55 (0.97)	3.61 (1.02)	3.74 (0.96)	4.82	**0.001**
Usability & Self-Efficacy	2.74 (0.82)	2.95 (0.79)	3.08 (0.88)	3.21 (0.84)	3.42 (0.83)	6.14	**< 0.001**
Attitudes Toward AI	3.55 (0.92)	3.61 (0.89)	3.68 (0.91)	3.65 (0.96)	3.71 (0.97)	1.93	0.106
Social Norms	2.98 (1.15)	3.04 (1.08)	3.09 (1.12)	3.21 (1.10)	3.38 (1.14)	3.27	**0.012**
Institutional Support	2.31 (1.08)	2.34 (0.98)	2.39 (1.05)	2.42 (1.02)	2.37 (1.07)	0.74	0.565

*SD *standard deviation. *F* statistics and *P*-values derived from one-way ANOVA. Bold *P*-values indicate statistical significance at *α* = 0.05. Post-hoc Tukey HSD comparisons revealed that 5th-year students scored significantly higher on usability and self-efficacy than 1st-year (*P* = 0.008) and 2nd-year (*P* = 0.014) students

Independent samples *t*-tests were conducted to examine gender differences across the five constructs (Table 7). No statistically significant differences were observed between male and female students for educational benefit (*P* = 0.631), usability and self-efficacy (*P* = 0.683), attitudes toward AI (*P* = 0.748), social norms (*P* = 0.832), or institutional support (*P* = 0.881). These findings suggest a broadly uniform perceptual profile across genders within this sample.

**Table 7:** Gender differences in construct means (independent samples *t*-test)

Construct	Female x̄ (SD)	Male x̄ (SD)	t	*P*	Public (*n* = 120)	Private (*n* = 175)	t	*P*
Educational Benefit	3.62 (0.98)	3.54 (1.03)	0.48	0.631	3.55	3.64	-0.89	0.374
Usability & Self-Efficacy	3.17 (0.84)	3.10 (0.87)	0.41	0.683	3.08	3.21	-1.32	0.188
Attitudes Toward AI	3.67 (0.93)	3.61 (0.98)	0.32	0.748	3.61	3.69	-0.76	0.448
Social Norms	3.14 (1.13)	3.09 (1.10)	0.21	0.832	3.08	3.18	-0.71	0.479
Institutional Support	2.36 (1.05)	2.33 (1.02)	0.15	0.881	2.29	2.42	-1.01	0.312

Comparisons by university type (public vs. private) did not yield statistically significant differences across constructs (all *P* > 0.05) (Table 7), indicating that AI-related perceptions are not institutionally stratified despite differences in resource environments.

### Correlation analysis between constructs

Table 8 presents both the correlation matrix and a structured theoretical interpretation of key relationships aligned with TAM and UTAUT assumptions. Pearson correlation coefficients were computed between all five Likert-scale constructs to examine the strength and direction of inter-construct relationships (Table 8). Educational benefit was most strongly correlated with attitudes toward AI (*r* = 0.56, *P* < 0.001) and moderately correlated with usability and self-efficacy (*r* = 0.48, *P* < 0.001) and social norms (*r* = 0.34, *P* < 0.001). The association between educational benefit and institutional support, while statistically significant (*r* = 0.22, *P* = 0.003), was notably weaker, suggesting that positive educational perceptions are sustained largely independently of institutional provision. Attitudes toward AI was significantly correlated with social norms (*r* = 0.51, *P* < 0.001), indicating that peer-level endorsement and individual attitudes are mutually reinforcing constructs in this sample. Institutional support yielded the weakest correlations across the matrix, consistent with its role as an external structural variable decoupled from students’ immediate perceptual experience of AI engagement.

**Table 8:** Pearson correlation matrix of likert-scale constructs with theoretical alignment (*n* = 295)

Construct	1	2	3	4	5
1. Educational Benefit	—				
2. Usability & Self-Efficacy	0.48^***^	—			
3. Attitudes Toward AI	0.56^***^	0.44^***^	—		
4. Social Norms	0.34^***^	0.29^***^	0.51^***^	—	
5. Institutional Support	0.22^**^	0.18^**^	0.13^*^	0.21^***^	—

**Theoretical Interpretation of Key Relationships**					
***Relationship***		***Interpretation***			
Attitudes ↔ Educational Benefit (*r* = 0.56)		Strong association consistent with TAM: attitudes function as a proximal determinant of perceived usefulness			
Usability ↔ Attitudes (*r* = 0.44)		Supports TAM assumption linking ease of use/self-efficacy to attitudinal formation			
Social Norms ↔ Attitudes (*r* = 0.51)		Reflects UTAUT social influence mechanism			
Institutional Support ↔ Educational Benefit (*r* = 0.22)		Weak facilitating conditions effect in this context			
Institutional Support ↔ Attitudes (*r* = 0.13)		Minimal influence of institutional structures on cognitive perceptions			

^*^ *P* < 0.05; ^**^ *P* < 0.01; ^***^ *P* < 0.001

All coefficients are Pearson’s *r*

Lower triangle is displayed for clarity

Correlation magnitudes are interpreted in relation to TAM and UTAUT theoretical expectations

The observed correlation pattern is directionally consistent with TAM and UTAUT assumptions, particularly the strong association between attitudes toward AI and perceived educational benefit. The moderate correlations between usability/self-efficacy and attitudes, as well as between social norms and attitudes, further support the theorised role of cognitive and social determinants in shaping technology-related perceptions.

### Hypothesis testing using regression analysis

#### Hypothesis evaluation

The regression results provide partial support for the proposed hypotheses.

H3 was supported, as attitudes toward AI emerged as the strongest independent predictor of perceived educational benefit (*β* = 0.38, *P* < 0.001; see Table 9). H1 and H2 received indirect support, as prior correlation analysis (Table 8) demonstrated significant positive associations between usability and self-efficacy (*r* = 0.44) and social norms (*r* = 0.51) with attitudes toward AI, consistent with their theorised antecedent roles. H4 was not supported, as institutional support did not demonstrate a statistically significant independent effect on perceived educational benefit (*β* = 0.08, *P* = 0.113; Table 9). H5 (mediation) could not be formally tested, as mediation analysis requires structural modelling; however, the observed pattern—strong associations between usability/self-efficacy and attitudes, and between attitudes and educational benefit—is consistent with a potential mediating role (see Tables 8 and 10). A standard MLR was conducted to identify the independent contribution of each construct to perceived educational benefit, with all predictors entered simultaneously (Table 9). The overall model was statistically significant (*F*(4, 290) = 52.4, *P* < 0.001) and accounted for 42% of the variance in educational benefit scores (*R²* = 0.42, adjusted *R²* = 0.41). Attitudes toward AI emerged as the strongest independent predictor (*β* = 0.38, *P* < 0.001), followed by usability and self-efficacy (*β* = 0.26, *P* < 0.001). Social norms also contributed significantly (*β* = 0.15, *P* = 0.002). Institutional support did not contribute a statistically significant independent effect (*β* = 0.08, *P* = 0.113). These findings are consistent with TAM and UTAUT expectations, particularly regarding the central role of attitudinal and usability-related constructs, and identify attitudes and critical self-efficacy as key leverage points for curricular intervention in this context.

**Table 9:** Multiple linear regression predicting perceived educational benefit (*n* = 295)

Predictor	Hypothesis Link	B	SE	β (std.)	T	*P*	95% CI	Interpretation
(Constant)		0.41	0.17	—	2.41	0.017	[0.08, 0.74]	
Attitudes Toward AI	H3	0.39	0.05	**0.38**	7.22	**< 0.001**	[0.29, 0.50]	Strong direct predictor of educational benefit
Usability & Self-Efficacy	H1, H5	0.30	0.06	**0.26**	4.48	**< 0.001**	[0.17, 0.43]	Likely indirect effect via attitudes
Social Norms	H2	0.17	0.05	**0.15**	3.13	**0.002**	[0.06, 0.28]	Social influence contributes indirectly
Institutional Support	H4	0.09	0.06	0.08	1.59	0.113	[− 0.02, 0.20]	No independent effect detected
Model fit		*R²* = 0.42		*Adj. R²* = 0.41		*F*(4,290) = 52.4	*P* < 0.001	
Metric			Value					
*ΔR²* (without attitudes)			0.29					
*ΔR²* contribution of attitudes			+ 0.13					

*B *unstandardised regression coefficient, *SE *standard error, *β *standardised coefficient, *CI *confidence interval. Bold *β* values indicate *P* < 0.05. Dependent variable: Educational Benefit subscale mean. Predictors entered simultaneously (standard entry). *VIF* values ranged from 1.12 to 1.68, indicating no problematic multicollinearity

**Table 10:** Approximate indirect effects (Baron & Kenny logic-based interpretation)

Path	Estimate	Interpretation
Usability → Attitudes	*β* ≈ 0.42	Substantial positive association
Attitudes → Benefit	*β* = 0.38	Strong predictive effect
Indirect Effect	≈ 0.16	Suggestive of moderate mediation
Social Norms → Attitudes	*β* ≈ 0.49	Substantial positive association
Indirect Effect	≈ 0.19	Suggestive of moderate mediation

The model demonstrated substantial explanatory power (*R²* = 0.42), indicating that the included predictors collectively account for a meaningful proportion of variance in perceived educational benefit. Diagnostic evaluation indicated no significant violations of normality or homoscedasticity assumptions.

Taken together, the group comparisons, correlation analysis, and regression modelling provide convergent evidence that students’ perceptions of AI in pharmacy education are shaped primarily by individual-level cognitive and experiential factors rather than institutional structures. While seniority and academic performance contribute modestly to variation, attitudinal and self-efficacy constructs consistently demonstrate the strongest associations with perceived educational benefit.

## Discussion

This study provides the first systematic characterisation of AI tool adoption, educational perceptions, and institutional context among pharmacy students in Syria. The findings cohere around a central paradox that has significant implications for both educational quality and patient safety: students are adopting AI-enabled learning practices with enthusiasm and at scale, in a structural environment that offers them neither guidance in how to use these tools critically nor protection from the professional risks that uninformed AI use entails. Understanding this paradox in its full dimensions requires attending to each of the five constructs examined and to the structural conditions that shape them. The 86.8% adoption rate documented in this study places Syrian pharmacy students among the highest-adopting populations reported in the international literature, comparable to figures observed in several prosperous, digitally saturated settings [1, 2]. This finding is counterintuitive given the documented infrastructure constraints of which 34.6% of participants reported that internet costs limited their AI use, only 1.4% held paid subscriptions, and a fifth of students lacked sufficient English proficiency to use most tools effectively. The apparent paradox resolves when AI adoption is understood not as a supplementary activity but as a compensatory one.

From a behavioural adaptation perspective, this pattern likely reflects the convergence of unmet informational needs, constrained educational infrastructure, and the exceptionally low activation energy required to engage with contemporary LLMs. In resource-constrained environments, students may preferentially adopt technologies that minimise time, financial expenditure, and dependence on institutional systems. Generative AI tools satisfy all three conditions simultaneously by providing immediate, conversational, and continuously available academic assistance. The dominance of ChatGPT (96.5%) which is a tool with no enrolment barriers and a capable Arabic-language interface, could reinforces this interpretation.

Equally important is the remarkably limited use of specialised evidence-oriented platforms such as Lexicomp, Elicit, and Semantic Scholar. This asymmetry suggests that students preferentially select highly accessible conversational systems over domain-specific evidence tools that require greater information literacy or formal training. Mechanistically, conversational LLMs reduce cognitive friction by collapsing search, interpretation, and synthesis into a single interactional interface, whereas specialised databases demand pre-existing competencies in evidence retrieval and critical appraisal. The resulting behavioural preference may inadvertently reinforce superficial fluency over evidence-based pharmacotherapeutic reasoning.

The structural conditions governing how tools are accessed, in what linguistic register, with what prior critical training, and against what institutional backdrop, fundamentally shape whether AI engagement constitutes a learning opportunity or a source of epistemic risk. The pattern of educational benefit perceptions is instructive beyond the aggregate mean. The highest-rated benefit — AI helping to understand difficult pharmacy topics more quickly ($\overline{x}$ = 3.82) — reflects the particular value of generative AI as an on-demand pedagogical scaffold: it can restate complex pharmacokinetic or mechanistic concepts in simplified language, generate analogies, and provide iterative clarification in ways that asynchronous textbook engagement cannot. The second-highest-rated benefit, improvement of study notes and summaries ($\overline{x}$ = 3.74), is consistent with well-documented LLM strengths in information condensation and reformatting. Both of these use cases are relatively low-stakes: errors in a simplified explanation of a receptor mechanism are more easily detected and corrected than errors in clinical dosing or interaction information. It is therefore analytically significant that AI enhancement of clinical case understanding received the lowest rating within the educational benefit subscale ($\overline{x}$ = 3.38), with the highest *SD* (1.40). Clinical reasoning additionally depends on contextual prioritisation, uncertainty management, and therapeutic trade-off analysis, cognitive processes that remain difficult for current LLM architectures to reproduce consistently. Unlike factual recall tasks, clinical pharmacotherapy problems frequently require probabilistic judgement and dynamic integration of incomplete patient information. This mismatch between probabilistic clinical reasoning and pattern-based language generation may partially explain students’ reduced confidence in AI-supported clinical learning. This indicates both lower perceived utility and greater heterogeneity of experience. Clinical case reasoning demands not only accurate factual recall but the integration of patient-specific variables, contraindication profiles, and therapeutic algorithms.

LLMs are known to generate syntactically coherent yet factually inaccurate clinical outputs, a phenomenon termed hallucination. The lower rating for clinical benefit may reflect students’ intuitive recognition that AI outputs in this domain are less reliable, or alternatively that the complexity of clinical reasoning tasks exposes the limits of AI-generated guidance in ways that simpler explanatory tasks do not. Either interpretation points toward clinical pharmacotherapy as the domain in which AI literacy training is most urgently needed and where the consequences of its absence are most consequential. The most theoretically and practically significant finding of this study is the pronounced intra-subscale divergence within the usability and self-efficacy construct. Students rated ease of learning and interface clarity positively ($\overline{x}$: 3.57 and 3.61), affirming that the interface-level accessibility of contemporary AI tools is genuinely high. Yet the two items measuring critical AI competence [troubleshooting incorrect outputs ($\overline{x}$ = 2.68) and evaluating information credibility ($\overline{x}$ = 2.75)] fell meaningfully below the scale midpoint. This divergence instantiates what might be termed an asymmetry between operational fluency and evaluative competence: students have sufficient skill to engage fluently with AI tools but insufficient evaluative capacity to calibrate their trust in the information those tools generate. The educational and professional hazards of this profile are substantial. A pharmacy student who encounters a hallucinated drug interaction in a ChatGPT response — described with the confident, well-structured prose that characterises LLM output — and who lacks the epistemic tools to cross-reference it against authoritative sources such as Lexicomp or Micromedex, may internalise that error as fact. Repeated over hundreds of study interactions, such errors may accumulate into a systematically distorted pharmacological knowledge base that surfaces only when it is tested in clinical practice. Mortlock and Lucas [4] characterised this dynamic as a compound risk in which academic integrity concerns are secondary to the more fundamental problem of information quality, and Weidmann [3] similarly argued that information verification competence must be treated as a non-negotiable outcome of pharmacy education in the AI era. The present data provide LMIC-specific empirical grounding for these arguments. It is also notable the low mean for the dependency item (2.15). It may signify that students do not perceive themselves as requiring much help to use AI or creates a specific kind of risk. Students who simultaneously feel confident in their AI use and are unable to evaluate the accuracy of AI outputs are precisely those least likely to seek verification or institutional guidance. This combination renders the absence of institutional scaffolding particularly dangerous: there is no metacognitive mechanism in place to trigger help-seeking behaviour in the students who most need it. The attitudes subscale returned the highest aggregate mean of the five constructs (3.66), with endorsement of AI in learning approaching the top of the scale ($\overline{x}$ = 3.95). This level of enthusiasm, while not unexpected given global trends in student AI adoption [2, 6, 13], demands careful contextual interpretation. The co-occurrence of high enthusiasm and low critical AI literacy does not represent a stable or educationally sound equilibrium. In the absence of structured frameworks for critical engagement, positive attitudes toward AI risk are becoming vectors of uncritical acceptance — a phenomenon consistent with what Ali [14] described as the deformation rather than the reshaping of pharmacy education. The moderate endorsement of concern that AI may displace effortful learning ($\overline{x}$ = 3.20) is one of the more interpretively rich findings in the dataset. This item captures what cognitive science literature refers to as automation bias. Automation bias refers to the cognitive tendency to reduce independent analytical effort when an intelligent system is available to perform a task. The fact that a majority of students endorsed this concern, even while reporting high AI use, suggests a capacity for metacognitive awareness that remains inactivated in the current institutional environment. Institutions that harness this existing ambivalence may be well positioned to redirect students’ natural enthusiasm into epistemically responsible practices.

The institutional support construct produced the starkest finding in the study: an $\overline{x}$ of 1.55 out of 5.00 for perceived university provision of AI resources, a value that approaches the theoretical floor of the scale and that was replicated across the near-unanimous reporting of absent guidance (93.6%) and training (95.3%). This finding reflects a profound institutional deficit in educational AI governance and student support infrastructure. The implications of this absence are multi-layered. First, in the absence of institutional policies, students have no normative framework within which to assess whether their AI use is academically appropriate or constitutes a form of academic dishonesty. The boundaries between legitimate AI assistance using an LLM to clarify a pharmacokinetic concept and academically compromised AI use are blurred in ways that vary by context, assignment type, and disciplinary expectation. Without explicit institutional guidance, students cannot be expected to navigate these boundaries reliably [4]. Second, the absence of institutional training forecloses the development of critical AI literacy competencies through formal educational channels, displacing this developmental task entirely onto informal, unsupervised self-learning. Third, and perhaps most significantly, the absence of institutional engagement signals to students that their AI practices are neither surveilled nor considered professionally significant, which may reduce the motivation for self-critical reflection.

While Issa et al. [6] observed that institutional readiness lagged behind adoption across several Arab countries, the magnitude of the infrastructural vacuum documented among Syrian institutions significantly exceeds regional averages, exposing an acute vulnerability in post-conflict educational tech governance. This may reflect the compound effect of conflict-related institutional disruption, resource scarcity, and regulatory inattention to educational technology governance factors that are not unique to Syria but that manifest with particular intensity in post-conflict settings. The findings thus extend the institutional support literature into a domain — conflict-affected LMIC pharmacy education, where the gap has previously been documented anecdotally but not empirically quantified. The digital access findings introduce a dimension that is absent from most analogous studies conducted in high-income settings. The combination of language barriers (22.0% insufficient English proficiency), cost barriers (34.6% internet cost-constrained), and near-universal reliance on free-tier tools creates a layered access inequality that limits not only whether students can use AI, but which tools they can use and how effectively. The moderate rating for Arabic-language tool availability (3.17 ± 1.45) reflects a landscape in which Arabic-language AI support exists but is heterogeneous and incoherently accessible such finding is consistent with the broader underrepresentation of Arabic in the training corpora of dominant LLMs. Students who lack English proficiency are, by this structural logic, confined to tools with weaker Arabic-language performance, generating outputs of lower quality and higher inaccuracy risk. This represents a compounding inequality among the students with the most constrained access are simultaneously exposed to the lowest-quality AI-generated information. Addressing this requires attention not only to institutional policy but to the language politics of AI development and a broader structural challenge that pharmacy educators in Arab-majority countries should collectively advocate for within national and regional educational policy frameworks. The correlation, year-of-study, and regression analyses strengthen this interpretation.

The behavioural mechanism driving this dynamic can be explained through the lens of cognitive resource allocation under severe material deprivation. When external institutional support approaches a functional floor, the administrative context ceases to serve as a facilitating condition or a normative barrier. Instead, technology acceptance collapses entirely into an individual compensatory cognitive strategy. The strong predictive power of attitudes (*β* = 0.38, *P* < 0.001) and usability (*β* = 0.26, *P* < 0.001) indicates that the student’s internal calculation of immediate cognitive payoff—specifically, the immediate reduction of study preparation friction—overrides any risk awareness regarding data credibility or algorithmic bias. The technology is accepted not because it is officially validated, but because its low-friction accessibility fills a critical institutional resource void.

The significance of social norms (*β* = 0.15, *P* = 0.002) — together accounting for 42% of variance (*R²* = 0.42) suggests that AI adoption within pharmacy education is not solely an individual cognitive process but also a socially reinforced behaviour. Peer-to-peer normalisation may accelerate technology diffusion independently of institutional endorsement, particularly in environments where formal governance is absent. In such contexts, informal student networks effectively become substitute regulatory ecosystems that shape acceptable AI practices, perceptions of utility, and thresholds of trust.

Institutional support predicted none of this variance (*β* = 0.08, *P* = 0.113), consistent with its near-zero correlations across the matrix (*r* = 0.13–0.22) and with the qualitative finding that 93.6% of students received no institutional guidance. One plausible explanation is that institutional structures in this context are functionally displaced by autonomous student adaptation strategies. Because students developed AI practices independently and prior to formal institutional engagement, perceived educational benefit may have become cognitively anchored to personal experimentation rather than organisational facilitation. Under such conditions, facilitating conditions cease to operate as primary determinants of perceived usefulness, diverging from classical UTAUT assumptions developed largely in stable, resource-abundant educational systems.

The year-of-study analysis reveals that whatever competence students gain is entirely self-generated: usability and self-efficacy climbed significantly across the five years (*F*(4,290) = 6.14, *P* < 0.001) while institutional support remained flat (*P* = 0.565), confirming that curricular intervention — not resource provision — is the lever that matters. The structural heterogeneity of the Institutional Support subscale is particularly salient in this respect: item IS1 (perceived university provision of AI resources, $\overline{x}$ = 1.55) and item IS2 (perceived availability of Arabic-language AI tools, $\overline{x}$ = 3.18) differ by 1.63 scale points in their means and are conceptually non-parallel—the former indexes institutional infrastructure provision and the latter indexes the adequacy of the AI tool ecosystem in a linguistic register relevant to Arabic-speaking students. The co-occurrence of high inter-item mean discrepancy and conceptual heterogeneity raises the possibility of low or near-zero inter-item correlation, which would produce unacceptably low or even negative *α* for this subscale. *CR* and *AVE* entail *λi* from CFA, derived from the item-level covariance structure; *HTMT* requires the full 17 × 17 inter-item Pearson correlation matrix, partitioned into hetero-trait (between-construct) and mono-trait (within-construct) blocks. Acceptable benchmarks for that analysis are *CR* ≥ 0.70 (acceptable) and *CR* ≥ 0.80 (good), *AVE* ≥ 0.50 for convergent validity, and *HTMT* < 0.85 for strict or < 0.90 for lenient discriminant validity, following established guidance for health professions education research.

Taken together, the findings suggest that Syrian pharmacy students have entered a phase of unsupervised AI integration characterised by rapid behavioural adoption, positive affective orientation toward AI, and insufficient development of evaluative safeguards. The coexistence of high operational fluency with low verification competence creates a structurally unstable educational equilibrium in which AI systems may progressively shape pharmacological understanding without corresponding growth in critical appraisal capacity. This imbalance may be particularly consequential in pharmacy education, where inaccuracies in drug-related reasoning possess downstream implications for medication safety and clinical decision-making. The collective evidence from this study supports a framework for institutional action organised across three levels. At the curricular level, explicit AI literacy modules should be integrated into core pharmacy courses, with content calibrated to the specific risk profile of pharmacy practice. Such modules should address the mechanics of LLM hallucination and its particular manifestation in pharmacological content; practical workflows for cross-referencing AI outputs against established drug information databases; academic integrity principles as applied to AI-assisted study and writing; and the appropriate use of specialist AI-enhanced tools such as Lexicomp, Elicit, and Semantic Scholar for evidence-based practice. These competencies should be assessed formally rather than left to informal development. Kattan et al. [5] and Aziz et al. [15] have both identified the absence of assessed AI literacy outcomes as a critical gap in pharmacy curriculum frameworks globally; the present data provide LMIC-specific empirical urgency for this argument.

This compensatory adoption pattern illustrates an unplanned instance of ‘educational leapfrogging’ within conflict-affected environments. Bypassing traditional evolutionary phases of institutional digital infrastructure—such as centralised enterprise databases, subscription-based medical libraries, and formal institutional portals—Syrian pharmacy students have directly integrated advanced generative models into their primary learning ecologies. While this leapfrogging effectively circumvents physical resource scarcities, it simultaneously strips away the foundational layers of source verification and institutional curation that typically safeguard the accuracy of professional clinical training.

At the policy and governance level, pharmacy schools in Syria and comparable settings must develop and disseminate clear, written policies governing AI use across the range of academic activities including; coursework, laboratory reports, clinical case assignments, and research training. These policies should be differentiated by use case rather than adopting blanket permissive or prohibitive stances, and should be revisited on an annual basis as the AI landscape evolves. The involvement of students in the development of these policies and building on the ambivalence and metacognitive awareness already evident in this sample and this may enhance both the quality and the legitimacy of the resulting frameworks.

At the infrastructure and equity level, institutional and national policymakers should prioritise investments in internet access quality, cost subsidisation for educational AI subscriptions, and the development and procurement of Arabic-language AI educational resources. The regional underdevelopment of Arabic-language AI tools is not merely a technical problem but an educational equity problem with direct implications for the quality of pharmacy workforce preparation across the Arab region. Regional pharmacy associations and educational accreditation, like the Syrian Ministry of Higher Education, are well-positioned to catalyse collective action in this domain in ways that individual institutions cannot accomplish in isolation. Future work in this area should be incorporated into decision-making bodies as well as monitoring AI’s fast development for up-to-date betterment in pharmacy education.

This study extends TAM and UTAUT scholarship by demonstrating that technology acceptance dynamics in conflict-affected and resource-constrained educational systems may differ substantially from those observed in stable high-resource environments. Specifically, the findings suggest that individual adaptive behaviours and self-directed digital competence can partially compensate for absent facilitating conditions, weakening the conventional predictive role of institutional support. This indicates that technology acceptance in disrupted educational ecosystems may operate through alternative pathways in which necessity-driven self-regulation supersedes formal organisational scaffolding. Future theoretical refinement of technology acceptance frameworks should therefore account for structural instability, infrastructural scarcity, and autonomous learner adaptation as context-sensitive modifiers of AI adoption behaviour.

### Limitations

Several methodological and structural limitations must be acknowledged when interpreting the current findings. First, the cross-sectional study design captures only a single temporal snapshot within a rapidly evolving AI landscape, preventing the evaluation of long-term behavioural changes or causal trajectories. Sampling was confined to an online convenience sample predominantly drawn from university students, which introduces substantial selection bias. This approach inherently excluded students suffering from severe infrastructural deprivations or total lack of internet access, thereby potentially overestimating general tool adoption rates and underestimating the true magnitude of localised socioeconomic and digital barriers. Furthermore, because the cohort was strictly limited to undergraduate pharmacy students within Syria, the generalisability of the findings to other health professions or alternative regional LMIC educational contexts is constrained. Second, important psychometric and modelling boundaries apply to the survey instrument. The *Attitudes Toward AI* subscale yielded a Cronbach’s *α* (*α* = 0.653) below the standard acceptability threshold due to conceptual heterogeneity introduced by directionally mixed items regarding learning ambivalence. Additionally, the *Social Norms* and *Institutional Support* subscales were structurally limited to two-item measures, which masks potential multi-dimensional variances; this is particularly evident given the extreme mean discrepancy observed between the infrastructure and linguistic components of institutional support. Methodologically, while the multi-variable linear regression model (LRM) confirmed robust relational dependencies (*R*^*2*^ = 0.42), the lack of structural equation modelling (SEM) precluded a formal, simultaneous verification of the latent measurement pathways and complex mediational systems hypothesised in the theoretical framework.

## Conclusion

This study establishes that undergraduate pharmacy students in a conflict-affected, resource-constrained environment have independently and systematically integrated generative artificial intelligence (AI) into their primary educational workflows. Operating as an autonomous, compensatory mechanism to offset severe institutional and infrastructural resource deficits, this high rate of adoption occurs entirely outside formal educational scaffolding or official policy boundaries. Sociologically, this sudden integration represents a form of educational leapfrogging, wherein students bypass traditional stages of institutional digital infrastructure. However, this self-directed transition introduces a profound epistemic vulnerability: the coexistence of positive learning attitudes alongside an unverified mastery of interface mechanics creates an illusion of clinical competence. Lacking the systematic evaluative literacy required to identify algorithmic hallucinations or cross-reference automated data against verified drug compendia, future pharmacy professionals face a significant risk of internalising plausible but clinically hazardous inaccuracies. Theoretically, these findings challenge classical technology acceptance models, such as the unified theory of acceptance and use of technology (UTAUT). In severely disrupted environments, facilitating conditions—specifically institutional support—become decoupled from perceived educational benefits. Rather than acting as a structural prerequisite for adoption, the complete vacuum of institutional support serves as the primary catalyst for individual, adaptive technology use. Consequently, it is recommended that national accreditation bodies and medical education policymakers move away from passive, defensive, or exclusionary technology policies. Curricular frameworks must immediately evolve to treat AI verification literacy not as an ancillary skill, but as a core component of digital health literacy and clinical safety. Future research must transcend cross-sectional descriptive surveys and implement longitudinal, multi-centred evaluative designs to systematically measure how structural AI literacy interventions protect clinical reasoning and safeguard patient-centred decision-making in low- and middle-income countries (LMICs).

## Supplementary Information

Supplementary Material 1.

## Data Availability

The datasets generated and/or analysed during the current study are available from the corresponding author upon reasonable request.

## Abbreviations

AI: Artificial intelligence
ANOVA: One-way analysis of variance
AVE: Average variance extracted
*β*: Path coefficient
CFA: Confirmatory factor analysis
CR: Composite reliability
HTMT: Heterotrait–monotrait ratio
LLM: Large language model
LMICs: Low- and middle-income countries
LRM: Linear regression model
MLR: Multiple linear regression
*R*^²^: Coefficient of determination
SD: Standard deviation
SEM: Structural Equation Modelling
TAM: Technology acceptance model
UTAUT: Unified theory of acceptance and use of technology
