# Supplementary material for: Adoption of artificial intelligence tools among pharmacy students in Syria: patterns of use, educational perceptions, and institutional barriers
Source: BMC Med Educ. 2026 Jun 17;26:1381. doi: 10.1186/s12909-026-09708-4 (PMC13508235; doi:10.1186/s12909-026-09708-4)
Supplement: Supplementary file 1 [file 12909_2026_9708_MOESM1_ESM.docx]

**Appendix I. Survey Instrument (English Translation)**

The original survey was administered in Arabic. The following is a complete English translation of all items as presented to participants, preserving the original section structure and item numbering. Response options are indicated in italics.

**SECTION 1: ELIGIBILITY AND CONSENT**

1. I confirm that I am 18 years of age or older and am currently enrolled as a pharmacy student in Syria.

*[Yes / No]*

2. Full name and phone number (optional — for researcher contact only).

*[Open text — or select: I prefer to participate anonymously / Confidential participation]*

3. I agree to participate in this study on an anonymous basis.

*[Yes / No]*

**SECTION 2: DEMOGRAPHIC AND ACADEMIC BACKGROUND**

4. Age (in years).

*[Open numeric field]*

5. Gender.

*[Female / Male / Prefer not to state]*

6. Type of university.

*[Public / Private]*

7. Name of university.

*[Open text field]*

8. Current year of study.

*[1st Year / 2nd Year / 3rd Year / 4th Year / 5th Year / Recent Graduate]*

9. Average monthly household income (USD equivalent).

*[Less than 500 USD / 500–1,000 USD / More than 1,000 USD]*

10. Academic performance during your studies (self-assessment).

*[Honors / Very Good / Good / Average]*

**SECTION 3: DIGITAL ACCESS AND INFRASTRUCTURE**

11. I have reliable internet connectivity for academic study purposes.

*[Yes / No]*

12. I have regular access to a computer or smartphone capable of running AI tools.

*[Yes / No]*

13. The monthly cost of internet service limits my ability to use AI tools frequently.

*[Yes / No]*

14. My English language proficiency is sufficient to use most AI tools effectively.

*[Yes / No]*

15. I hold a paid monthly subscription to an AI tool.

*[Yes / No / Free tier only]*

**SECTION 4: AI USAGE PATTERNS**

16. Have you used any AI tools for academic purposes during the past three months?

*[Yes / No]*

17. How frequently do you use AI tools for pharmacy-related academic tasks?

*[Daily / Several times per week / Weekly / Monthly / Rarely]*

18. Which AI tools do you primarily use? (Select all that apply.)

*[ ] ChatGPT — general-purpose conversation, learning, and writing*

*[ ] Google Translate — text and website translation*

*[ ] DeepL — precision translation for academic and research texts*

*[ ] Microsoft Translator — instant multilingual translation*

*[ ] Microsoft Copilot — conversation and productivity assistance*

*[ ] Canva AI — AI-powered design and presentation creation*

*[ ] Semantic Scholar — intelligent academic literature search*

*[ ] Elicit — AI-assisted summarisation of scientific papers and research questions*

*[ ] Scite — scientific citation verification*

*[ ] Notion AI — smart note organisation and intelligent writing assistance*

*[ ] Grammarly — grammar and academic writing quality checking*

*[ ] Lexicomp — clinical drug interaction and medication information*

*[ ] Micromedex — advanced clinical drug information database*

*[ ] Drugs.com Interaction Checker — rapid drug interaction screening*

*[ ] Replika — emotional support and social interaction*

*[ ] Other (please specify): _______________________________*

19. What academic tasks do you routinely perform using AI tools? (Select all that apply.)

*[ ] Summarising articles and academic texts*

*[ ] Explaining difficult concepts*

*[ ] Creating personalised study plans*

*[ ] Generating practice and self-assessment questions*

*[ ] Searching for drug information*

*[ ] Generating diagrams and visual study aids*

*[ ] Solving and reviewing past examination questions*

**SECTION 5: INSTITUTIONAL CONTEXT**

20. I have received formal institutional guidance from my faculty regarding the responsible use of AI tools.

*[Yes / No]*

21. I have received formal training on how to use AI tools effectively in my learning.

*[Yes / No]*

22. Based entirely on personal effort, I have taught myself to use AI tools.

*[Yes / No]*

**SECTION 6: PERCEPTIONS OF AI IN PHARMACY EDUCATION**

Please indicate your level of agreement with each of the following statements using the scale: 1 = Strongly Disagree, 2 = Disagree, 3 = Neutral, 4 = Agree, 5 = Strongly Agree.

***Subscale A: Educational Benefits***

23. AI helps me understand difficult pharmacy topics more quickly.

*1 2 3 4 5*

24. AI improves the quality of my study notes and summaries.

*1 2 3 4 5*

25. Using AI tools increases my readiness for examinations.

*1 2 3 4 5*

26. AI has strengthened my understanding of different clinical cases.

*1 2 3 4 5*

27. Exposure to AI tools helps me prepare for future careers in pharmacy.

*1 2 3 4 5*

***Subscale B: Usability and AI Self-Efficacy***

28. Learning to use AI tools has been easy for me.

*1 2 3 4 5*

29. I find AI tools clear and easy to understand.

*1 2 3 4 5*

30. I require a great deal of help in order to use AI tools. *

*1 2 3 4 5*

31. I am able to identify and correct problems when AI outputs appear to be incorrect.

*1 2 3 4 5*

32. I am able to evaluate the credibility of information generated by AI tools.

*1 2 3 4 5*

***Subscale C: Attitudes Toward AI in Learning***

33. In general, using AI for learning is a good idea.

*1 2 3 4 5*

34. I enjoy using AI tools while studying.

*1 2 3 4 5*

35. I am concerned that AI tools may replace the core effort of learning.

*1 2 3 4 5*

***Subscale D: Social Norms***

36. My peers encourage me to use AI tools for studying.

*1 2 3 4 5*

37. I encourage my peers to use AI tools for studying.

*1 2 3 4 5*

***Subscale E: Institutional Support***

38. My university provides the resources necessary for using AI tools.

*1 2 3 4 5*

39. The AI tools I need are available in Arabic or provide Arabic-language support.

*1 2 3 4 5*

** Item 30 is reverse-coded for construct scoring purposes. Items 23–39 constitute the five-subscale Likert battery (Section 6); all other items are categorical or open-response. The instrument was originally developed and administered in Arabic.*
